# Editorial review of qualitative and quantitative characteristics of the lumbar multifidus muscles: Comparison of the magnetic resonance imaging and musculoskeletal ultrasound

**DOI:** 10.1016/j.inpm.2025.100555

**Published:** 2025-03-10

**Authors:** Lynn Kohan

**Affiliations:** Department of Anesthesiology, University of Virginia Health System, USA

Nonspecific low back pain (NSLBP) is a common but heterogenous diagnosis. While lumbar muscle degeneration has been a known causative factor in the development of NSLBP for some time, recently there has been an increased interest in the role that multifidus muscle (MM) dysfunction may play in this condition [1]. It remains somewhat unclear if MM dysfunction is the cause or the effect of NSLBP. MM act as the core stabilizers of the spine. Dysfunction in core stabilization and associated changes in muscle morphology either through inactivity or secondary to direct muscle injury, infection, inflammatory disease, or nerve injury is thought to contribute to the development on NSLBP. A systematic review found that the smaller cross-sectional area of the MM was predictive of low back pain [2]. Additionally, degenerative structural changes to the MM have been found to predict worse outcomes and impair recovery in patients with NSLBP [3,4]. Falla et al. also suggested that NSLCP can affect muscle structure by altering muscle function [5].

Both macroscopic and microscopic changes in the MM structure have been observed in patients with NSLBP including a decreased in cross-sectional area (CSA) and increase in fat content of the MM [6,7]. Evidence is mixed regarding microscopic changes with some studies suggesting increased content of type IIX (fast twitch glycotic fibers as compared to type I (slow twitch oxidative) [8], while other studies failing to show changes in muscle fiber type [9].

Differing imaging modalities can be used to investigate the changes in muscle morphology including ultrasound (US), magnetic resonance imaging (MRI), and computed topography (CT). US and MRI have generally been the favored modalities since CT scans have poor ability to differentiate soft tissue types and thus may not adequately access muscle characteristics [2,10,11]. MRI has the advantage of directly showing adipose tissue, which enables the ability to detect even subtle variations in both muscle morphology and tissue composition [12]. Three major signs of muscle degeneration detected on imaging include decreased cross sectional area (CSA), increase in radiographic density, and an increase in fat deposits [13]. The CSA of the L4-5 vertebral space has been reported to be affected 6–9 times more frequently than any other spinal level [14].

NSLBP can present similarly clinically regardless of etiology/pain mechanism however different etiologies require different specific targeted treatment approaches. Developing mechanisms by which to further elucidate these etiologies can enhance patient care. Gofeld et al.‘s study is of particular interest as it adds to current literature investigating diagnostic imaging modalities that may help to better subclassify patients with NSLBP secondary to MM atrophy, which ultimately may lead to appropriate targeted treatment and better pain related outcomes. Prior to the current study, a direct comparison between the efficacy of MRI versus US in investigating MM atrophy had not been performed.

Gofeld et al. measured CSA and fatty infiltration of the MM to evaluate muscle pathology in their investigation. The purpose of their investigation was to compare the accuracy and reliability of US compared to MRI in evaluating the CSA and degree of fatty infiltration (using the Kjaer system) of the MM in patients with CNLBP. The authors took measurements at the most symptomatic level as well as L2 (control level).

The authors found a strong correlation in degree of muscle fatty deposits, noted by similar Kajier scores between MRI and US at the painful levels as compared to L2 where the correlation was found to be “fair”. This difference may be secondary to the decreased thickness and hydration of the MM which may have been interpreted as atrophy at more cranial levels.

The authors noted a poor correlation between CSA between US and MRI. The authors postulated that US may perform more poorly in this measurement due to a number of factors including the challenge of achieving a strict axial view as minor cranial or caudal tilts could result in an artificially inflated CSA. In a prior study, Russo et al. also reported that US can provided some information on MM thickness, but failed to conclude that this measurement could reliably predict treatment outcomes [15]. The authors concluded that US therefore, may not be a reliable tool to assess for CSA.

While not the main objective of their study, it is of interest that the authors did not report any positive prone instability test findings amongst patients with NSLBP and MM fatty infiltration. Currently, the PIT test is used to assess for lumbar spinal instability which may be a consequence of MM atrophy and is being used as one tool in the selection criteria for patients to be treated with MM stimulation, however studies are not conclusive regarding its reliability [16,17]. More research is warranted to clarify the clinical relevance the PIT test has in identifying patients with NSLBP secondary to MM atrophy.

Tenderness to palpation of the lumbar paraspinals muscles is generally thought to be non-specific, however has been found to be a predictor of successful outcomes following lumbar medial branch radiofrequency ablation [18]. Gofeld and team found the degree of tenderness to lumbar paraspinal palpation to correspond to the highest degree of MM atrophy, thus cautiously suggestive as having a role in identifying patients with NSLBP caused by MM atrophy.

Thus, while small, this study adds additional insight into diagnostic modalities that may help to identify patients with NSLBP secondary to MM atrophy with MRI likely remaining a superior tool to US at this time.

## Declaration of competing interest

I receive institutional funding for research from FUSMobile, Vertex, Averitas, and Avanos.

I have provided consultative services for Avanos.
